# Editorial: New technological devices for dermatological application: upgrades and efficacy

**DOI:** 10.3389/fmed.2024.1491435

**Published:** 2024-09-18

**Authors:** Gislaine Ricci Leonardi, Isabel F. Almeida, Marlus Chorilli

**Affiliations:** ^1^School of Pharmaceutical Sciences, Universidade Estadual de Campinas (UNICAMP), Campinas, Brazil; ^2^UCIBIO—Applied Molecular Biosciences Unit, Laboratory of Pharmaceutical Technology, Faculty of Pharmacy, University of Porto, Porto, Portugal; ^3^Associate Laboratory i4HB, Institute for Health and Bioeconomy, Faculty of Pharmacy, University of Porto, Porto, Portugal; ^4^School of Pharmaceutical Sciences, São Paulo State University—UNESP, Araraquara, Brazil

**Keywords:** skin, dermatological devices, ultrasound, microneedles, drug delivery, skin images, skin science

The skin is the body's largest organ and plays a crucial role in maintaining our overall health and wellbeing. Advances in technology have provided a wealth of new tools and techniques for studying and treating skin conditions, from innovative imaging devices to novel drug delivery systems. As we deepen our understanding of the skin, non-invasive instruments and imaging devices are becoming increasingly important to aid in the treatments of skin conditions and helping to prevent and minimize signs of skin aging. Pequeno and Bagatin summarized the importance of ultrasound analysis in monitoring topical treatments for skin rejuvenation. High-frequency ultrasound has a crucial role in assessing skin aging through various parameters, such as skin thickness, subepidermal low echogenicity band (SLEB) characterization, and echogenicity assessment. This analysis can help guide more personalized interventions for each patient and assess the effectiveness of cosmetics and procedures (1). The scientific literature, through studies using high-frequency ultrasound, has shown that topical formulations with ascorbic acid 5% or tretinoin 0.05% provide a reduction in SLEB (2, 3). High-frequency ultrasound imaging is a real-time, important current device for the diagnosis, clinical management and therapy monitoring of skin conditions. It is also a valuable tool for assessing the performance of cosmetic and dermatological products.

Another technique that has emerged as a key tool for advancing skin science is reflectance confocal microscopy (RCM). Guerra et al. have introduced an innovative RCM-based method to assist in the quantification of skin cell transformation. It is well-established that actinic keratosis (AK) is a precancerous lesion associated with excessive sun exposure. Understanding the cellular changes that occur in actinic keratosis is crucial for comprehending the development of the disease and for monitoring the effectiveness of treatments. RCM is a modern and technological method of evaluating the structure of the epidermis, with the advantage of not requiring biopsies or invasive procedures (4). Guerra et al. show that RCM is a very important modern technique for providing visual microscopic information about the subclinical stages of AK, allowing the monitoring of disease progression and the assessment of treatments efficacy through disease regression. The study reported in this RT provides a photographic scale to guide dermatologists in identifying the various signs of cellular transformation, which could be highly beneficial for early diagnosis, and evaluating the effectiveness of proposed treatments.

This Research Topic also presents two case studies using new technological devices for dermatological application. Song et al. highlighted the use of superficial radiotherapy in the treatment of keloids, through a literature review and case study. The use of radiotherapy in the treatment of keloids has been described since the beginning of the twentieth century, associated with surgical procedures to reduce scar formation or alone to induce scar atrophy. Recently, computerized technologies have enhanced the targeting of x-rays, allowing for precise control over their accuracy and intensity. This advancement minimizes harmful effects on the body and improves their application in therapies, such as keloid treatment. Superficial radiotherapy is a type of external radiotherapy used to treat diseases close to the surface of the skin, characterized by low penetrating power. It is typically administered using low-energy X-rays (photons) or electrons. The literature reports several studies about the use of superficial radiation in the treatment of keloids and skin cancer (5–7). In the present study, the authors observed the use of superficial radiation associated with trephination, shrinkage and flattening of hypertrophic keloid keloids in a boy with congenital syndactyly that cannot be treated by surgical excision.

Kishimoto et al. reported the use of 755-nm picosecond Alexandrite laser therapy on atypical henna-induced Riehl's melanosis. Picosecond lasers have very short individual pulse durations. In this way, they can be used to effectively and safely remove different types of pigmented lesions in a shorter period. Its mechanism of action is based on the rupture of pigment particles into very small fragments, which are phagocytosed and removed by macrophages (8, 9). An alexandrite laser uses an alexandrite crystal as a laser source producing a specific wavelength of light in the infrared spectrum, which is 755 nm, and is considered a red light laser. This laser works on the principle of photothermolysis for the treatment of a selected area (8). Riehl's melanosis can be defined as a disease characterized by numerous fine or reticulate, acquired macules of pigmentation of uncertain etiology (10). In this study, Kishimoto et al. reported safe and effective treatment of henna-induced-atypical Riehl's melanosis using a 755-nm picosecond Alexandrite laser. Immunohistochemical analyses revealed a potential role of CD8-positive lymphocytes in henna-induced inflammation and hyperpigmentation of the basal layer and a role of melanophages in the pigmented dermis of Riehl's melanosis.

The application of Artificial Intelligence (AI) in medicine has garnered enormous attention over the past decade (11, 12). A plethora of AI tools (13) has been proposed for dermatological applications, many of which use AI algorithms for skin cancer classification (14). Primiero et al. proposed a protocol to validate AI-based tools to perform a holistic analysis of the whole patient using Total Body Photography (TBP) rather than the dermoscopic image of a single lesion. The goal of these tools is to assist clinicians in monitoring melanoma and perform patient risk profiling.

The proposed protocol describes the methods to construct a comprehensive dataset for machine learning based on patient data, including TBP, medical history and genetic risk factors. Algorithms developed with these datasets will address several clinically relevant gaps that previous skin lesion classifier algorithms often lack.

This Research Topic reflects recent innovations in technological devices for dermatological applications, which are expected to continue to evolve based on technological advances and the improved maturity of AI tools.

## References

[1] Bagatin E Caetano LdVN Soares JLM. Ultrasound and dermatology: basic principles and main applications in dermatologic research. Expert Rev Dermatol. (2013) 8:463–77. 10.1586/17469872.2013.838513

[2] Vergilio MM Aiello LM Furlan AS Caritá AC Azevedo JR Bolzinger MA. *In vivo* evaluation of topical ascorbic acid application on skin aging by 50 MHz ultrasound. J Cosmet Dermatol. (2022) 21:4921–6. 10.1111/jocd.14892 35238148

[3] Sumita JM Miot HA Soares JLM Raminelli ACP Pereira SM Ogawa MM. Tretinoin 005% cream versus 5% peel for photoaging and field cancerization of the forearms: randomized, evaluator-blinded, clinical trial. J Eur Acad Dermatol Venereol. (2018) 32:1819–26. 10.1111/jdv.15020 29704456

[4] Longo C Casari A Beretti F Cesinaro AM Pellacani G. Skin aging: *in vivo* microscopic assessment of epidermal and dermal changes by means of confocal microscopy. J Am Acad Dermatol. (2013) 68:e73–82. 10.1016/j.jaad.2011.08.021 22000768

[5] Nestor MS Berman B Goldberg D Cognetta Jr AB Gold M Roth W. Consensus guidelines on the use of superficial radiation therapy for treating nonmelanoma skin cancers and keloids. J Clin Aesthet Dermatol. (2019) 12:12–8. 30881578 PMC6415702

[6] McGregor S Minni J Herold D. Superficial radiation therapy for the treatment of nonmelanoma skin cancers. J Clin Aesthet Dermatol. (2015) 8:12–4. 26705443 PMC4689506

[7] Liu EK Cohen RF Chiu ES. Radiation therapy modalities for keloid management: a critical review. J Plast Reconstruct Aesthet Surg. (2022) 75:2455–65. 10.1016/j.bjps.2022.04.099 35817711

[8] Zawodny P Wahidi N Zawodny P Duchnik E Stój E Malec WR. Evaluation of the efficacy of the 755 nm picosecond laser in eliminating pigmented skin lesions after a single treatment based on photographic analysis with polarised light. J Clin Med. (2024) 13:304. 10.3390/jcm13020304 38256438 PMC10816936

[9] Zhou Y Hamblin MR Wen X. An update on fractional picosecond laser treatment: histology and clinical applications. Lasers Med Sci. (2023) 38:45. 10.1007/s10103-022-03704-y 36658259 PMC9852188

[10] Kumarasinghe SPW Pandya A Chandran V Rodrigues M Dlova NC Kang HY. A global consensus statement on ashy dermatosis, erythema dyschromicum perstans, lichen planus pigmentosus, idiopathic eruptive macular pigmentation, and Riehl's melanosis. Int J Dermatol. (2019) 58:263–72. 10.1111/ijd.14189 30176055

[11] Rabbani N Kim GYE Suarez CJ Chen JH. Applications of machine learning in routine laboratory medicine: current state and future directions. Clin Biochem. (2022) 103:1–7. 10.1016/j.clinbiochem.2022.02.011 35227670 PMC9007900

[12] Davenport T Kalakota R. The potential for artificial intelligence in healthcare. Fut Healthc J. (2019) 6:94–8. 10.7861/futurehosp.6-2-94 31363513 PMC6616181

[13] Alomary SA Mackenzie E Khan S Rao BK. Mobile apps in dermatology: bridging innovation and care. Skin Res Technol. (2024) 30:e13640. 10.1111/srt.13640 38424722 PMC10904765

[14] Salinas MP Sepúlveda J Hidalgo L Peirano D Morel M Uribe P. A systematic review and meta-analysis of artificial intelligence versus clinicians for skin cancer diagnosis. NPJ Digit Med. (2024) 7:125. 10.1038/s41746-024-01103-x 38744955 PMC11094047

